# Hematopoietic Prostaglandin D Synthase Inhibitor PK007 Decreases Muscle Necrosis in DMD *mdx* Model Mice

**DOI:** 10.3390/life11090994

**Published:** 2021-09-21

**Authors:** Sai Yarlagadda, Christina Kulis, Peter G. Noakes, Mark L. Smythe

**Affiliations:** 1 School of Biomedical Sciences, Faculty of Medicine, The University of Queensland, Brisbane, QLD 4072, Australia; sai.yarlagadda@uqconnect.edu.au; 2 Institute for Molecular Bioscience, The University of Queensland, Brisbane, QLD 4072, Australia; c.kulis@imb.uq.edu.au (C.K.); m.smythe@imb.uq.edu.au (M.L.S.)

**Keywords:** PGD2, HPGDS, inflammation, regenerating muscle fibers, Duchenne muscular dystrophy (DMD), muscle creatine kinase (CK-MM), *mdx*

## Abstract

Duchenne muscular dystrophy (DMD) is characterized by progressive muscle weakness and wasting due to the lack of dystrophin protein. The acute phase of DMD is characterized by muscle necrosis and increased levels of the pro-inflammatory mediator, prostaglandin D2 (PGD2). Inhibiting the production of PGD2 by inhibiting hematopoietic prostaglandin D synthase (HPGDS) may alleviate inflammation and decrease muscle necrosis. We tested our novel HPGDS inhibitor, PK007, in the *mdx* mouse model of DMD. Our results show that hindlimb grip strength was two-fold greater in the PK007-treated *mdx* group, compared to untreated *mdx* mice, and displayed similar muscle strength to strain control mice (C57BL/10ScSn). Histological analyses showed a decreased percentage of regenerating muscle fibers (~20% less) in tibialis anterior (TA) and gastrocnemius muscles and reduced fibrosis in the TA muscle in PK007-treated mice. Lastly, we confirmed that the DMD blood biomarker, muscle creatine kinase activity, was also reduced by ~50% in PK007-treated *mdx* mice. We conclude that our HPGDS inhibitor, PK007, has effectively reduced muscle inflammation and fibrosis in a DMD *mdx* mouse model.

## 1. Introduction

Duchenne muscular dystrophy (DMD) affects approximately 1 in 3500 males and is characterized by progressive muscle weakness and wasting due to the lack of dystrophin protein [1]. A deficiency in dystrophin results in the degeneration of skeletal and cardiac muscle [2,3]. Degeneration of these muscles occurs as early as four years of age in patients, with initial signs of disease being limb muscle weakness or abnormal gait [4,5]. As the child ages, symptoms range from Bower’s sign, a sign that indicates muscle weakness of the proximal limb muscles, to swollen calves due to fat and fibrotic tissue build-up within the gastrocnemius muscle [3,5].

Dystrophin has a critical role in stabilizing the cell membrane of muscle and protecting the myofibers from disruptions during contraction [6]. A malfunction in this protein can trigger breaks in the sarcolemma, particularly when tension is applied on these cells. With breaks in sarcolemma, the degradation of damaged muscle fibers is initiated. Additionally, muscle creatine kinase (CK-MM), a validated biomarker for muscle damage in DMD which is located inside muscle fibers, escapes into the bloodstream and is a known DMD blood biomarker [7,8]. Initially, the body’s natural reaction is to regenerate these damaged fibers, however, over time the capacity to renew muscle fibers is depleted and fat or fibrotic tissue are used instead. As a result, these fat and fibrotic tissues weaken muscles [9]. These pathological events lead to chronic activation of the innate immune system, including inflammation [7].

Inflammation is a signal-mediated response to cellular stress caused by infectious agents, toxins, or physical stress, and in DMD, it is the result of weakened muscles due to fat and fibrotic tissue build-up [10]. Inflammation can be distinguished into two phases, acute and chronic [10]. The acute phase is classified as the body’s immediate response to an infection or injury and is initiated by the movement of white blood cells (mast cells, neutrophils, eosinophils, or basophils) to the inflammatory site [11,12]. In DMD, inflammation in the acute phase is primarily caused by the activation of the innate immune system. Activation of the innate immune system causes an over recruitment of pre-immune cells to the site of muscle damage. Immune cells detect this debris of tissue and generate pro-inflammatory and anti-inflammatory factors such as prostaglandins (PGs). PGs upregulate cell signaling proteins (e.g., tumor necrosis factor alpha (TNF-α)) and muscle degradation pathways (chronic inflammation) and suppress cell growth factors such as insulin-like growth factor-1 (IGF-1), which, in turn, causes greater skeletal tissue damage, enhancing the progression of DMD and ultimately muscle fibrosis [10,13].

Prostaglandins (PGs) are lipid autacoids derived from arachidonic acid [14]. PGs sustain homeostatic functions and facilitate pathogenic mechanisms, including the inflammatory response [11,15,16]. Four primary PGs are ubiquitously generated: prostaglandin D2 (PGD2), prostaglandin E2 (PGE2), prostaglandin F2α (PGF2α), and prostacyclin (PGI2). They act as autocrine and paracrine lipid mediators to maintain local homeostasis in the body [11,17]. During acute inflammation in DMD, the role for each prostaglandin becomes specific to maintain muscle tissue integrity. PGE2 is known to induce muscle stem cell expansion during muscle cell regeneration [17]. PGF2α regulates second stage muscle cell fusion by increasing the number of nuclei within myotubes and mediates regeneration, improving myonuclear accretion in the nascent myotube [7]. PGI2 reduces cell proliferation, inhibits cell growth during inflammation, which is beneficial in stopping fibroblasts from dividing, hindering the progression of DMD [18]. By contrast, PGD2 is a pro-inflammatory mediator produced by mast cells in DMD patients and is known to aggravate inflammation, causing profound tissue damage in skeletal muscle in patients suffering from muscular dystrophies [19].

Hematopoietic prostaglandin D synthase (HPGDS) is a sigma class glutathione-S-transferase family member found in peripheral tissues [16]. It is a cytosolic protein with a primary task of catalyzing the conversion of PGH2 to PGD2. In DMD patients, HPGDS expression has been detected, and shown to increase in both skeletal muscles and inflammatory cells such as mast cells during the early onset of the DMD, when muscle is beginning to undergo necrosis [16,19]. Previous studies in *mdx* mice reported that the production of PGD2 by HPGDS aggravates inflammation and causes profound muscle damage [14]. Further, these investigators demonstrated that muscle necrosis and PGD2 production were significantly decreased in *mdx* mice by inhibiting HPGDS production with HQL-79, a low 20 µM HPGDS inhibitor [14,16,19,20,21,22,23,24,25]. Here, we evaluate a newly designed inhibitor of HPGDS, PK007, which is 100-fold more potent than HQL-79 (see Supplementary Figure S1), in the *mdx* mouse model of DMD.

*Mdx* mice are the most used animal model in DMD studies due to the resemblance of initial DMD pathogenesis and physiology to humans [16,26]. In *mdx* mice, an acute onset of pathogenesis occurs at approximately 3 weeks of age. The acute phase in *mdx* mice is thought to be equivalent to 4–6 years in DMD boys [16,27]. This period is characterized by the presence of necrotic foci, newly regenerated nucleated myofibers, and high plasma concentrations of muscle creatine kinase [28]. Cyclical regeneration of muscles is a multi-step process including the activation and proliferation of muscle satellite (stem) cells, repair and maturation of damaged muscle fibers and connective tissue formation [29]. This cycle peaks between weeks 3 and 4, and this early phase is thought to model DMD in humans [30]. Detailed analysis of *mdx* muscle pathology by previous researchers reveals that hindlimb muscles such as the gastrocnemius and tibialis anterior are severely affected in the early phase of muscular dystrophy [31].

This study hypothesized that by selectively blocking the synthesis of PGD2, we will ameliorate DMD muscle pathology, resulting in improved muscle strength during the acute phase of DMD. The aim of this project was to validate the effect of the novel HPGDS inhibitor PK007 in the pathogenesis of *mdx* mice.

## 2. Materials and Methods

### 2.1. Animals

Three-week-old male C75BL/10 ScSn-*mdx* (*n* = 12) and C75BL/10 ScSn (strain control; *n* = 12) mice were used in this study, sourced from the Animal Resources Centre (ARC) Perth, WA Australia. Standard treatment guidelines for *mdx* mice were used throughout this study [31,32]. Mice were randomly allocated into two groups (*n* = 6) and were housed in individually ventilated cages (6 pups per cage). These cages were labeled A, B, C, or D and were treated in a double-blind manner with vehicle (0.5% methyl cellulose, 0.1% Tween80, and MilliQ water), or HPGDS inhibitor (PK007: 10 mg/kg/day in 0.5% methylcellulose, 0.1% Tween80, and MilliQ water) via oral gavage daily. The mice were then monitored to ensure no adverse events occurred due to the administration of PK007. General health (eating, locomotion, and behavior), appearance (ruffled fur, depression, and animal movement) and weight loss were measured using ‘score sheets’ for the entire 10 days of treatment daily. Mice were scored a numerical value of 0–3, with 0 highlighting no change in the animal’s behavior and a score of 3 indicating adverse changes in the animal for each indicator described above (see Supplementary Figure S2). We observed no adverse events in *mdx* treated with either PK007 or vehicle. In addition, we also screened PK007 for potential off-target interactions in the Safety Screen44TM panel (Eurofin Cerep-Panlabs, Taiwan, China). PK007 was screened at 10 µM in this in vitro pharmacological screen for its potential to interfere with the binding of native ligands of 44 different receptors, ion channels, and enzymes, including COX-1 and COX-2. The results showed that PK007 does not have high binding to any targets and that there are no major safety issues (Supplementary Table S1).

### 2.2. Muscle Grip Strength Assessments

Hindlimb grip strength was measured daily, 2 h after oral gavage over the 10-day treatment period and was assessed using the IMADA^®^ grip device by the same individual for the duration of the study. The instrument measured the highest force generated by each mouse over the course of 5 trials over a 1 min cycle. The maximum force (N) produced over the trials was selected. This methodology followed TREAT-DMD standard operating procedures that have been published for the pre-clinical assessment of drugs (standard operating procedure (SOP) ID: DMD_M.2.2.001; [32]).

### 2.3. Muscle Dissection and Histochemical Staining

At the conclusion of the study, postnatal (p) 28 days (p28) (SOP ID: DMD_M.1.2.007; [32]), mice were euthanized via cervical dislocation. The gastrocnemius (GA) and tibialis anterior (TA) were dissected and snap frozen in liquid nitrogen and stored at −80 °C. For pathology, muscles dissected and immersed fixed in 4% paraformaldehyde–phosphate-buffered saline pH 7.4 (PBS) overnight at 4 °C. Muscles were then washed in PBS, dehydrated (70% ethanol, 90% ethanol, 100% ethanol (2×), and xylene (3×)), and processed into paraffin blocks. Transverse sections were cut at 7 µm using a Lecia RM 2245 microtome and were collected onto Super Frost Plus microscopic slides. These slides were stained with Mayer’s hematoxylin and eosin (H&E) (0.1%) and toluidine blue (acetate) stains (0.1% pH = 2.3). The stained slides were digitally imaged using a Leica Aperio slide scanner at 20× magnification for quantitative analysis. For quantitative analysis of regenerating muscle fibers, the following SOP was applied: DMD_M.1.2.007 [32], where muscle fibers with the nuclei in the center were considered ‘regenerating’. Following SOP DMD_M.1.2.007 [32], muscle fibrosis was determined by the presence of infiltrating cells and degenerating myofibers with fragmented sarcoplasm, as per previous studies [33]. The entire cross-sectional area of muscle fibrosis was then calculated, and the extent of fibrosis was reported as a percentage of the total muscle cross-sectional area.

### 2.4. Blood Collection and Muscle Creatine Kinase (CK-MM) Assay

After cervical dislocation, blood was collected from the heart, placed into cryovials, centrifuged at 4000 rpm for 10 min at 4 °C, and serum collected. A colorimetric creatine kinase activity assay kit was used to determine CK-MM levels (Abcam, Melbourne, Vic. Australia Cat. No.: ab155901). To determine optimal diluent concentration for the assay, a standard curve was created and then samples were run in duplicate and were measured at OD 450 nm on a Tecan M1000 Pro Plate Reader in a kinetic mode, every 1 min for 40 min at 37 °C.

### 2.5. Statistics

GraphPad Prism (Version 9.2.0 for Windows, GraphPad Software, La Jolla California USA, www.graphpad.com—accessed on 1–13 July 2021) was used for statistical analyses and a summary of each figure’s result is presented in Supplementary Table S2. All data were assessed for distribution using Shapiro–Wilk tests. To determine statistical significance between groups that assumed normal distribution, parametric tests were employed. Body weight data was analyzed using a two-way analysis of variance (ANOVA) with Greenhouse–Geisser correction (used to assess for a continuous outcome (i.e., body weight)). Hindlimb grip strength and histological analysis were analyzed using a two-way ANOVA with Tukey’s post hoc test (used to assess every mean with every other mean across the 4 treatment groups). CK-MM analysis was conducted by a 2-tailed unpaired *t*-test. Probability ‘*p*’ < 0.05 was considered significant. All values are presented as the mean ± standard error of the mean (SEM).

## 3. Results

### 3.1. No Change in Mean Body Weight over the 10-Day Treatment Period for Both Treated and Untreated Groups and WT Groups

**Figure 1:**
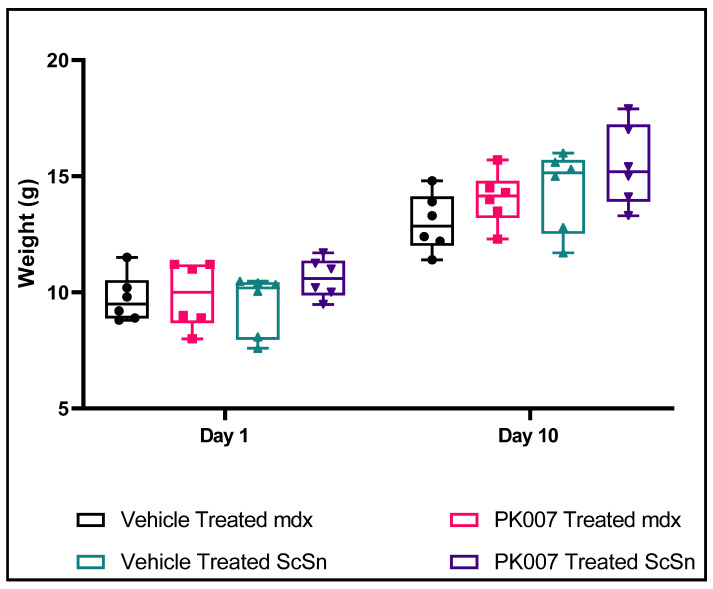
No significant change in mean body weight over the day 1 and day 10 treatment period for treated and untreated *mdx* and strain control (WT) mice. Figure 1 displays a box and whisker plot, highlighting the weight of both PK007-treated and vehicle-treated groups for both *mdx* and strain control (WT) mice on day 1 and day 10. *Mdx* vehicle- and PK007-treated groups displayed an average weight of 9.73 g ± 1.02 g and 9.88 g ± 1.42 g, respectively, on day 1. Strain control (WT) vehicle- and PK007-treated groups exhibited an average grip strength of 9.49 g ± 1.30 g and 10.61 g ± 0.85 g, respectively, on day 1. By day 10 *mdx* vehicle- and PK007-treated groups displayed an average grip weight of 13 g ± 1.24 g and 14.0 g ± 1.73 g, respectively, on day 10. Strain control (WT) vehicle- and PK007-treated groups exhibited an average weight of 14.0 g ± 1.73 g and 15.45 g ± 1.74 g, respectively, on day 10. Biological *n* = 6 and *p* > 0.05 using a two-way ANOVA Tukey’s post hoc test (*n* = 6). ± represent SEMs.

Starting body weights were similar for both treated and untreated *mdx* and C57Bl/10ScSn (strain control) mice (day 1, Table 1 and Figure 1). By the end of the study, both treated and vehicle groups of the mdx genotype had increased their body weights by ~70% (i.e., 9.73 g day 1 to 13.0 g day 10 for mdx vehicle, and 9.88 g day 1 to 14.05 g day 10 for mdx treated: Table 1). The treated and untreated strain control groups had also increased their body weights by similar amounts (i.e., by ~66–70%; 10.61 g day 1 to 15.45 g day 10 for strain control-treated, and 9.49 g day 1 to 14.40 g day 10 for strain control-vehicle; Table 1). Overall, there was no significant difference in body weight among the four groups (*p* > 0.05 and *n* = 6) and no adverse signs for standard behavioral parameters (Supplementary Figure S1) were observed during the study duration.

### 3.2. Mdx Mice Display Improved Muscle Grip by the End of Treatment with PK007

**Figure 2:**
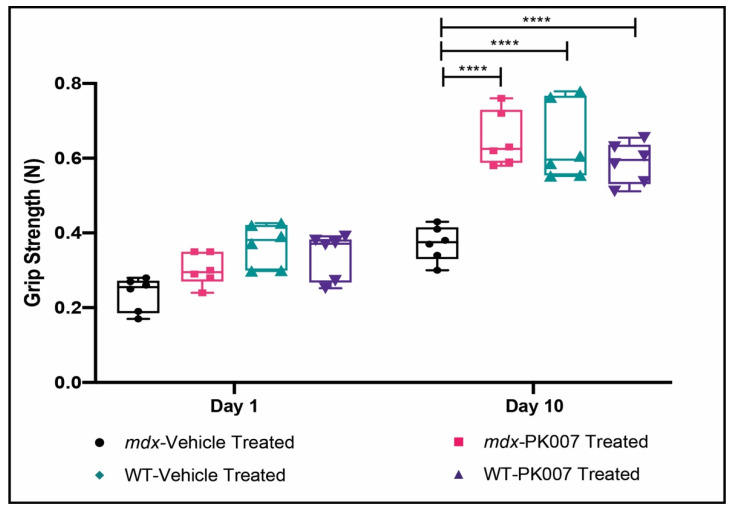
PK007-treated *mdx* mice display a higher grip strength compared to vehicle-treated *mdx* mice and similar grip strengths to strain control mice. Figure 2 displays a box and whisker plot, highlighting the grip strength of both PK007-treated and vehicle-treated groups for both *mdx* and strain control (WT) mice on day 1 and day 10. *Mdx* vehicle- and PK007-treated groups displayed an average grip strength of 0.237 N ± 0.019 N and 0.302 N ± 0.017 N, respectively, on day 1. Strain control (WT) vehicle- and PK007-treated groups exhibited an average grip strength of 0.368 N ± 0.023 N and 0.340 N ± 0.025 N, respectively, on day 1. By day 10 *mdx* vehicle- and PK007-treated groups displayed an average grip strength of 0.372 N ± 0.019 N and 0.650 N ± 0.030 N, respectively, on day 10. Strain control (WT) vehicle- and PK007-treated groups exhibited an average grip strength of 0.640 N ± 0.042 N and 0.587 N ± 0.022 N, respectively, on day 10. **** denotes *p* < 0.0001 at day 10. *p* values were calculated using a two-way ANOVA with Tukey’s post hoc test (*n* = 6).

Hindlimb muscle strength of the *mdx* vehicle-treated and *mdx* PK007-treated mice at treatment day 1, displayed an average grip strength of 0.237 N and 0.302 N, respectively (Figure 2 and Table 2). The strain control vehicle-treated and PK007-treated groups exhibited similar grip strengths to PK007-treated *mdx* cohort with average grip strengths of 0.368 N and 0.340 N, respectively (Figure 2 and Table 2). By treatment day 10, *mdx* PK007-treated group exhibited a significant two-fold increase (*p* < 0.0001) in grip strength (0.650 N), compared to the vehicle-treated group (0.372 N) (Figure 2 and Table 2). The PK007-treated *mdx* group displayed similar grip strengths to the strain control vehicle and PK007-treated groups (0.640 N and 0.587 N). These results indicate that our HPGDS inhibitor restored the dystrophy-associated strength deficit in *mdx* mice to similar levels seen in strain control mice.

### 3.3. PK007-Treated mdx Group Exhibits Fewer Regenerating Muscle Fibers in the TA and GA

**Figure 3:**
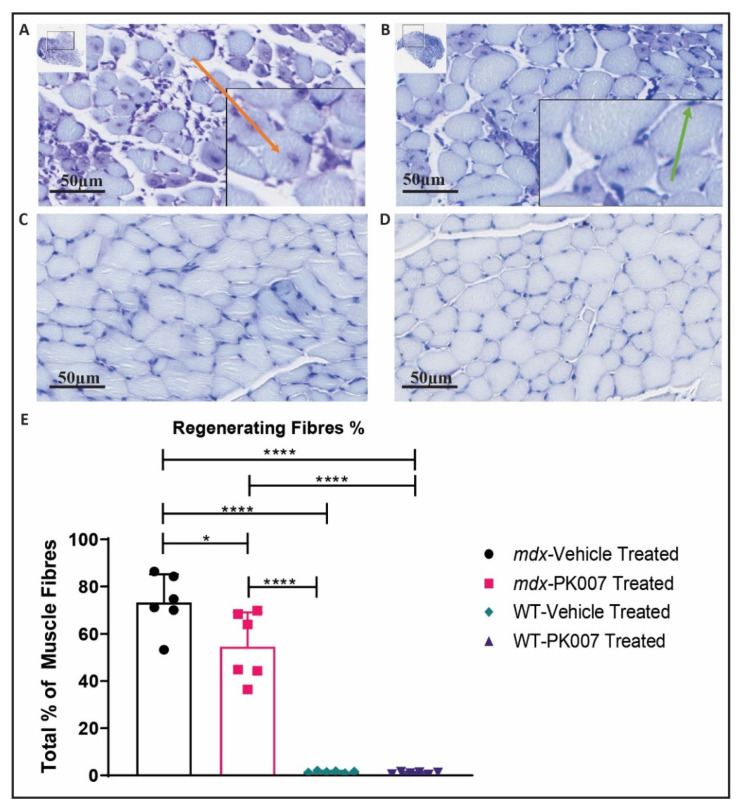
Toluidine blue stain of TA muscle highlighted a greater percentage of mature muscle fibers and fewer regenerating muscle fibers in the PK007-treated *mdx* group than the vehicle-treated *mdx* group. (**A**) A cross-sectional image of vehicle-treated *mdx* mice (p28). (**B**) A cross-sectional image of PK007-treated *mdx* mice (p28). (**C**) A cross-sectional image of vehicle-treated strain control mice (p28). (**D**) A cross-sectional image of PK007-treated strain control mice (p28). Scale bars = 50 µm. (**E**) The means and their respective SEMs (error bars) of the number of TA regenerating muscle fibers for *mdx* and strain control (WT) vehicle (untreated), PK007 (treated) groups. *Mdx* vehicle- and PK007-treated groups displayed an average percentage of regenerating muscle fibers of 73.29% ± 4.89% and 54.66% ± 5.89%, respectively. Strain control (WT) vehicle- and PK007-treated groups exhibited minimal regenerating muscle fibers at 1.60% ± 0.16% and 1.07% ± 0.18%. * Represents *p* = 0.0353 and **** represents *p* < 0.0001. *p* values were calculated using a 2-way ANOVA with Tukey’s post hoc test with *n* = 6.

**Figure 4:**
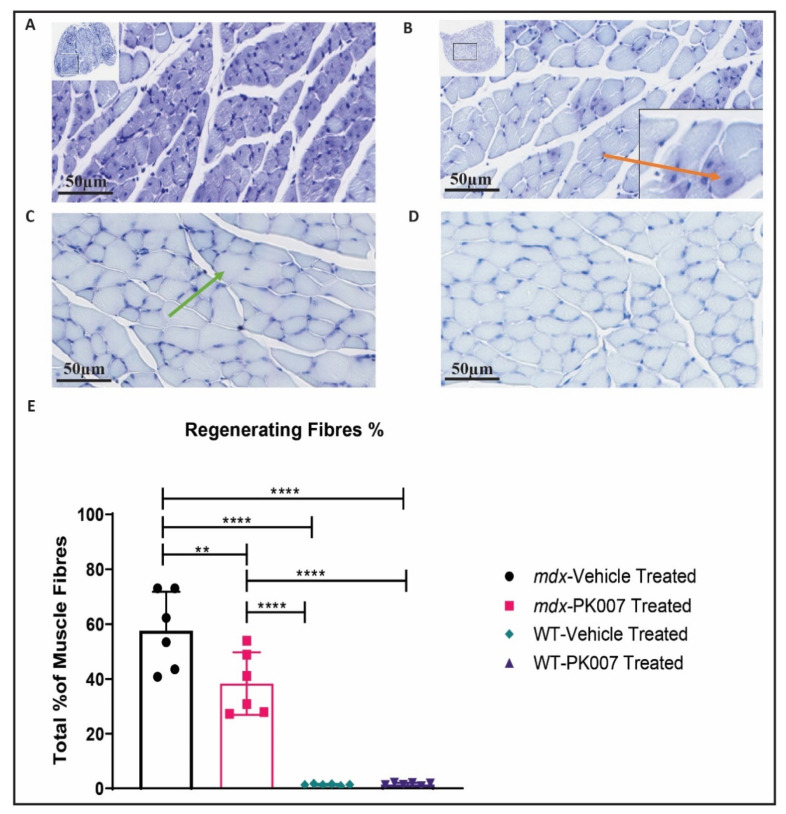
Toluidine blue stain of GA muscle highlighted a greater percentage of mature muscle fibers and fewer regenerating muscle fibers in the PK007-treated *mdx* group than the vehicle-treated *mdx* group. (**A**) A cross-sectional image of vehicle-treated *mdx* mice (p28). (**B**) A cross-sectional image of PK007-treated *mdx* mice (p28). Red arrow represents a regenerating muscle fiber. (**C**) A cross-sectional image of vehicle-treated strain control mice (p28). Green arrow depicts a mature muscle fiber. (**D**) A cross-sectional image of PK007-treated strain control mice (p28). Scale bars = 50 µm. (**E**) The means and their respective SEMs (error bars) of the percentage of GA regenerating muscle fibers from *mdx* and strain control (WT) vehicle (untreated), PK007 (treated) groups of mice. *Mdx* vehicle- and PK007-treated groups displayed an average percentage of regenerating muscle fibers of 57.72% ± 5.78% and 38.33% ± 4.66%, respectively. Strain control (WT) vehicle- and PK007-treated groups exhibited minimal regenerating muscle fibers at 1.38% ± 0.12% and 1.54% ± 0.21%. ** Represents *p* = 0.0036 and **** represents *p* < 0.0001. *p* values were calculated using a two-way ANOVA with Tukey’s post hoc test with *n* = 6.

The pathology of the TA and GA muscles was analyzed from cross-sections taken mid-way between their respective origin and insertions (mid-muscle), stained with toluidine blue or H&E stain (see Methods). The toluidine blue stain was used to assess the numbers of mature muscle fibers versus regenerating muscle fibers (Figure 3 and Figure 4). Regenerating muscle fibers were defined by their nuclei being present in the center of the muscle fiber (e.g., orange arrow in Figure 3A and Figure 4B; SOP ID: DMD_M.1.2.007; [32]). By contrast, mature muscle fibers were identified and defined as possessing nuclei located beneath the muscle’s plasma membrane (i.e., peripherally located nuclei [32]; green arrows in Figure 3B and Figure 4C).

We analyzed the numbers of regenerating muscle fibers for the fast twitch TA muscle using a toluidine blue stain (Figure 3). Referring to Figure 3E, the percentage of regenerating muscle fibers in the *mdx* PK007-treated group displayed fewer (~20% decrease) regenerating muscle fibers (54.66%) by comparison to the vehicle-treated group (73.29%; Figure 3E). As expected, the strain control mice displayed very few regenerating fibers (1.60% and 1.07%, Figure 3E). Overall, the *mdx* PK007-treated group had less muscle damage compared to its respective vehicle group (Figure 3).

Next, we quantified the percentage of regenerating muscle fibers in GA mid-muscle for both *mdx* and strain control mice. Referring to Figure 4E, for the percentage of regenerating muscle fibers, *mdx* PK007-treated mice displayed significantly fewer (~20% decrease) regenerating muscle fibers (38.33%), compared to *mdx* vehicle-treated groups which had a higher regenerating muscle fiber percentage (57.72%). Collectively, our TA and GA findings clearly demonstrate that PK007 treatment significantly decreased the level of muscle damage in *mdx* mice—during their ‘acute’ phase of DMD.

### 3.4. TA Muscles from PK007-Treated Mice Display Lower Levels of Muscle Fibrosis Compared to TA Muscles from mdx Vehicle-Treated Mice

**Figure 5:**
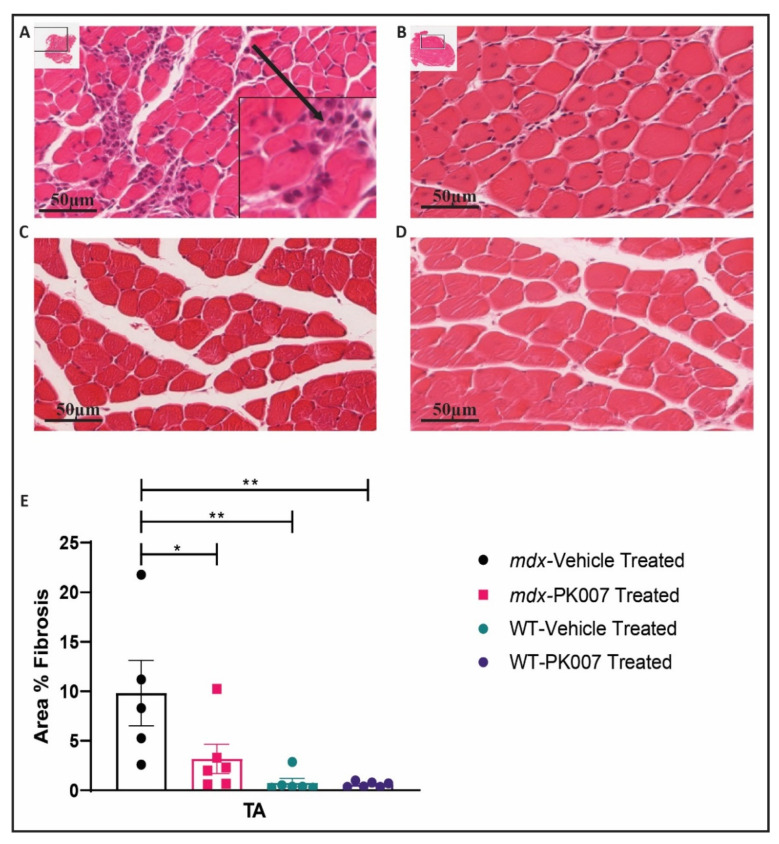
TA muscles from vehicle-treated *mdx* mice show greater fibrosis compared to TA muscles from PK007-treated mice. (**A**) A cross-section of the TA muscle of vehicle-treated *mdx* mice stained with hematoxylin and eosin at p28. Black arrow in the inset represents evidence of muscle fibrosis, characterized by the presence of single nuclei cells (inflammatory and fibroblast cells) replacing muscle fibers. (**B**) A comparable section from a PK007-treated *mdx* mouse at p28, showing fewer fibrotic cells. (**C**) A cross-sectional image of vehicle-treated strain control mice (p28). (**D**) A cross-sectional image of PK007-treated strain control mice (p28). Scale bars = 50 µm. (**E**) The mean area of muscle fibrosis which was quantified by selectively measuring the area of accumulated inflammatory and fibroblast cells with respect to the total cross-sectional area of muscle, expressed as a percentage. *Mdx* vehicle- and PK007-treated groups displayed an average cross-sectional area of 9.82% ± 7.41% and 3.12% ± 3.60%, respectively. Strain control (WT) vehicle- and PK007-treated groups exhibited minimal muscle fibrosis at 0.77% ± 1.03% and 0.59% ± 0.27%. Error bars = SEM. * Represents *p* = 0.0376 and ** represents *p* = 0.0025 and 0.0020. *p* values were calculated using a two-way ANOVA with Tukey’s post hoc test with *n* = 5 for *mdx* vehicle-treated group and *n* = 6 for all other groups.

**Figure 6:**
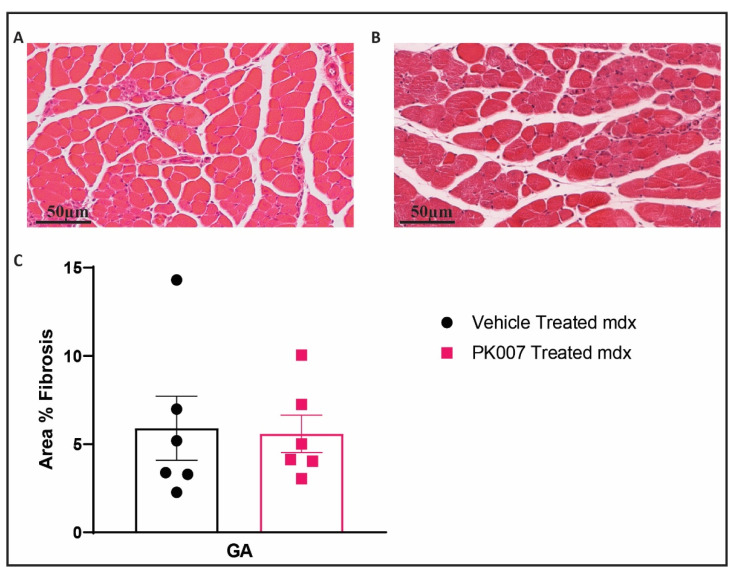
GA muscle displayed no significant muscle fibrosis in the untreated group of an *mdx* mouse model. (**A**) Cross-section of the GA muscle of vehicle-treated *mdx* mice (p28) stained with hematoxylin and eosin. (**B**) Comparable section from a PK007-treated *mdx* mouse (p28). (**C**) The total area % of muscle fibrosis which was quantified by selectively measuring the area where inflammatory cells and degenerating myofibers with fragmented sarcoplasm were present. *Mdx* vehicle- and PK007-treated groups display the average cross-sectional area, 5.90% ± 4.44% and 5.58% ± 2.61%, respectively. Error bars = SEM and scale bars = 50 µm. *p* = 0.8826 and was calculated by an unpaired *t*-test with *n* = 6.

The early (acute) phase of DMD is characterized by increased accumulation of extracellular matrix components such as collagens produced by proliferating fibroblasts whose proliferation is induced by invading immune cells, in regions of muscle damage (collectively termed fibrosis) [32]. We therefore examined and quantified the extent of fibrosis at the mid-section of TA and GA muscle across the PK007-treated and vehicle-treated *mdx* and strain control mice. TA muscle from *mdx* mice treated with PK007 displayed significantly less muscle fibrosis (3.12%) when compared to TA muscles from *mdx* vehicle-treated mice (9.82%; Figure 5). By contrast, when we examined the GA muscles from PK007-treated and vehicle-treated *mdx* mice, we observed low levels of fibrosis and the difference between the two treatment groups (5.90% and 5.58%) was not significant (*p* = 0.8826; Figure 6).

### 3.5. PK007 Treatment Reduces Serum Muscle Creatine Kinase (CK-MM) in mdx Mice

**Figure 7:**
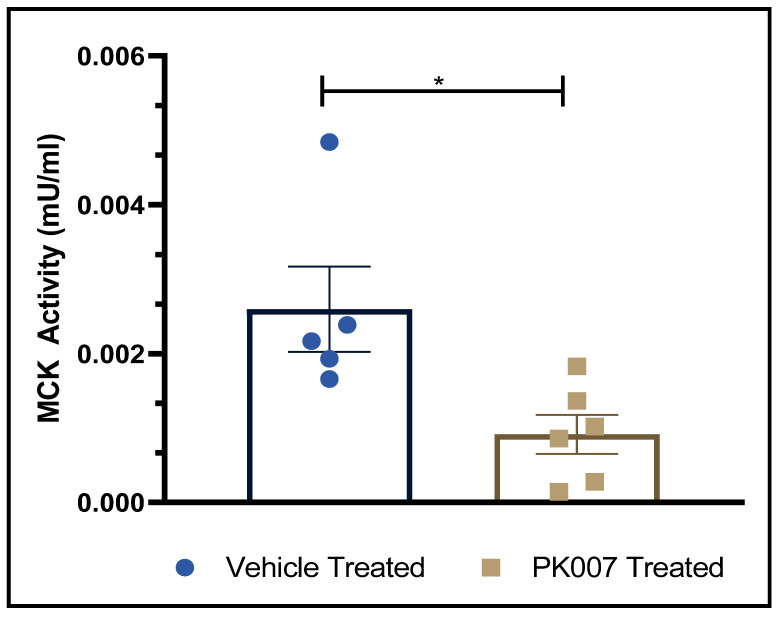
Muscle creatine kinase levels are reduced in the blood from PK007-treated *mdx* mice compared to vehicle-treated *mdx* mice. Shown are the mean and SEM values of blood creatine kinase levels, detected using a colorimetric CK-MM assay. Vehicle-treated and PK007-treated groups of *mdx* mice showed mean CK-MM levels of 0.002705 mU/mL and 0.001094 mU/mL, respectively. This study did not get an opportunity to assess the levels of WT CK-MM. * Denotes that *p* = 0.0196 which was calculated by an unpaired two-tailed *t*-test. Error bars represent SEM. Biological *n* = 5 for vehicle-treated group and *n* = 6 for *mdx* PK007-treated group.

We observed reduced levels of muscle damage (fibrosis) and fewer regenerating muscle cells in *mdx* mice treated with PK007 compared to vehicle-treated *mdx* mice, so we wanted to test if this translated into a lower level of CK-MM in the blood of PK007-treated *mdx* mice compared to vehicle-treated *mdx* mice. CK-MM in the blood of *mdx* mice and DMD boys has been shown to be a biomarker of muscle damage [20]. Serum CK-MM activity was prepared from the blood and analyzed using a colorimetric assay (see Methods). Vehicle-treated mice displayed an average CK-MM level of 0.0026 U/mL with the highest and lowest readings calculated at 0.0048 mU/mL and 0.0016 mU/mL, respectively. By contrast, the PK007-treated group displayed mean CK-MM levels of 0.0009 mU/mL of with the highest and lowest readings of 0.0018 mU/mL and 0.0001 mU/mL, respectively (Figure 7). These results showcase a ~50% increase of CK-MM in the muscle cells in *mdx* PK007-treated mice, indicating less muscle damage in this cohort.

## 4. Discussion

This study aimed to access the therapeutic efficacy of our HPGDS inhibitor PK007 in the treatment of the DMD acute phase, in *mdx* mice. This aim was achieved by following standard operating procedures (SOPs) for the pre-clinical assessment of drugs used in *mdx* mice to assess their therapeutic efficacy (see [32]; Neuromuscular Network, Treat NMD.org). Our results show that PK007 does significantly improve the level of muscle grip during the early acute phase of DMD in these mice (the muscle death phase between postnatal days 18 and 28). After 28 days in PK007-treated *mdx* mice, muscle grip was two-fold higher when compared to vehicle-treated *mdx* mice. This was supported by our pathological and biochemical analyses of hind limb muscle at the end of treatment. We show that hind limb muscles from PK007-treated *mdx* mice had ~20% fewer regenerating muscle fibers (55%) when compared to vehicle-treated *mdx* mice (73%; Figure 3). In other words, 20% of muscle fibers were saved from cell death during the acute phase of DMD in *mdx* mice, a percentage of rescue that resulted in improved muscle grip to the same levels as in strain control (wild type) mice (PK007 and vehicle treated). For TA muscles, we also showed that fibrosis was significantly reduced in PK007-treated *mdx* mice compared to TA muscles from vehicle-treated *mdx* mice. These findings were further corroborated by our biochemical analyses of CK-MM, which is released into the blood following muscle damage/death. We showed a ~50% decrease in CK-MM levels in blood from the PK007-treated *mdx* mice.

### 4.1. PK007 Improves Muscle Grip in mdx Mice

Current treatment options for DMD include corticosteroids, such as prednisolone, which inhibit the upstream effectors of the arachidonic pathway COX-1/2 [34]. Inhibition of COX-1/2 not only inhibits pro-inflammatory PGD2—the immediate product of HPGDS, but, importantly, the inhibition of anti-inflammatory prostaglandins such as PGE2 and PGF2α [11]. Hence, it is perhaps not surprising that most corticosteroids are not effective in increasing muscle strength during the acute phase of muscle damage in DMD [35,36]. One study conducted by Anderson et al. used the corticosteroid deflazacort to treat muscle weakness in *mdx* mice (acute phase; [31,37]). In this study, there was increased muscle regeneration resulting in a modest increase in muscle forelimb grip (~15%, [31,37]). By contrast, inhibition of HPGDS with PK007 did significantly increase muscle strength by ~50%, along with presence of more mature muscle fibers during the acute phase of DMD in *mdx* mice. Our findings add further support to the therapeutic value in selectively inhibiting the PGD2 pathway in DMD (acute phase). Furthermore, PK007 (IC_50_: 0.2 μM) is 100-fold more potent than HQL-79 (IC_50_: 20 μM) in an in vitro HPGDS inhibitor screening assay (Cayman Chemical, Ann Arbor, Michigan, USA, Item No. 600007) and is potent for binding HPGDS (Supplementary Figure S1).

PK007 also appears selective and safe (Supplementary Table S1), showing no significant binding in a Safety Screen44TM panel (Eurofin Cerep-Panlabs, Taiwan, China); namely, PK007 was assayed for its potential to interfere with the binding of native ligands of 44 different receptors, ion channels, and enzymes (including COX-1 and COX-2). The results show that PK007 does not have high binding to any targets underpinning known safety consequences. We also investigated the pharmacokinetics of PK007 in male Sprague Dawley rats following a single oral dose at 10 mg/kg. PK007 plasma concentration peaked at 1 h post-dose, suggesting rapid absorption, and had a half-life of 11 h (Supplementary Figure S3). This improved potency led to more significant reductions in muscle damage and fibrosis (discussed below) over previous HPGDS inhibitors in the *mdx* model. The SOPs for DMD animal model studies were followed, ensuring our results are directly comparable to previously published studies (see [16,38]).

### 4.2. Mdx Mice Treated with PK007 Results in a Greater Amount of Mature Muscle Fibers, Fewer Regenerating Muscle Fibers, and Decreased Percentage of Fibrosis in the TA Muscle

In our study, we observed a greater amount of regenerating muscle fibers in the TA compared to the GA in untreated (vehicle) *mdx* mice, indicating muscle damage was greater in the TA compared to the GA during this early acute phase of DMD; a finding supported by previous research [39,40,41]. This reduction in regenerating muscle and increase in mature muscle fibers in the *mdx* PK007-treated muscles could infer a mechanistic change in pathogenesis. A plausible explanation based on published literature is that if PGD2 production was reduced via PK007, a decrease in cytokine expression such as IL-6 and TNF-α can ensue [16]. As a result, recruitment of pre-immune cells (macrophages, TH2 and mast cells) could be decreased [42]. The suppressed activation of the innate immune system results in lower levels of inflammation and, thus, less muscle damage on the periphery. This would result in fewer satellite cells proliferating into regenerating muscle fibers and maintaining a greater percentage of mature muscle fibers in the TA muscle of the *mdx* PK007 group [30,43]. In our study, we observed a novel effect in which a greater percentage of mature muscle fibers remains in the TA muscle of *mdx* mice after PK007 treatment in the acute phase.

### 4.3. PK007 Treatment of mdx Mice Results in Decreased Fibrosis in TA Muscles but Not GA Muscles

The early (acute) phase of DMD is also characterized by increased accumulation of dividing fibroblasts whose proliferation is induced by invading immune cells, in regions of muscle damage (collectively termed fibrosis; [32,44]). In *mdx* mice, we observed significant levels of fibrosis in the TA muscle. Upon PK007 treatment, the levels of fibrosis were dramatically reduced. This observation supports previous research, where HQL-79, which also inhibits HPGDS, reduced muscle fibrosis in the lower limb muscles of *mdx* mice [16]. It is therefore likely that inhibition of HPGDS by PK007 has, via reduced PDG2 levels [42], resulted in reduced recruitment of immune cells into regions of damaged muscle, and consequently decreased muscle fibrosis [45]. Our results are consistent with this proposed mechanism; further studies will be required to validate this idea.

### 4.4. Decreased CK-MM Levels in PK007-Treated mdx Mice Indicate Lower Levels of Muscle Damage

CK-MM is known to be released from damaged sarcoplasmic reticulum into the blood of DMD patients and in *mdx* mice [32,44,45,46], and is an established biomarker of muscle damage. [45,47]. In our study, we observed a significant reduction in CK-MM levels in the blood of *mdx* mice treated with PK007, which further supports our idea that inhibition of HPGDS reduces muscle damage in *mdx* mice, a notion reinforced by reduced muscle pathology (reduced fibrosis and reduced levels of regenerating muscle) in the TA muscles of *mdx* mice treated with PK007 (discussed above) [32,48].

### 4.5. Limitations and Future Directions

In this study, we have shown that PK007 significantly improves muscle histology and grip strength. PK007 is a potent and selective inhibitor of HPGDS, suggesting the therapeutic effect is related to blocking PGD2 production. In future studies, it will be necessary to measure PGD2 levels in muscle tissue in *mdx* mice before and after treatment with PK007, and also measure other biochemical markers (such as TNF-α and IL-6; [9,14,48]), to further support the mechanism of action. It would also be ideal to examine the cellular expression and localization of other molecular markers of skeletal muscle damage such as HSP27 and dystrophin [49] beyond the indirect markers of muscle damage such as CK-MM.

It is well known that the *mdx* model’s pathogenesis is mild by comparison to human DMD [50,51]. In future, we would like to validate our findings in a more severe model of DMD, such as the new *mdx* strain developed by Jackson laboratories (strain name: DBA/2J-*mdx* and stock number: 013141) [52] This strain is reported to show an aggressive acute phase of the disease and a stronger cardiac phenotype in the chronic phase of DMD [52]. While we attained success in the acute phase of DMD in alleviating muscle necrosis, it is vital that we explore the long-term effects of PGD2 inhibition and its effect in the cardiac and respiratory system, as these are vital organs that depreciate in the chronic phase of DMD [53]

## 5. Conclusions

Based on the findings presented, our novel compound PK007 shows promise in treating the acute phase of DMD. It was further highlighted that by inhibiting HPGDS, we saw a 2-fold increase in hind limb grip strength, muscle protected from damage (20% decrease in damaged muscle fibers), lowered muscle fibrosis, and reduced CK-MM plasma levels (~50%) in the mdx PK007-treated group, suggesting that blocking HPGDS could provide an alternative therapeutic option in treating DMD.

## Figures and Tables

**Table 1:** No significant change in mean body weight over the 10-day treatment period for treated and untreated *mdx* mice. Shown are the daily mean weights in grams (g) for PK007-treated, vehicle-treated *mdx* mice, PK007-treated control, and vehicle-treated control mice. Biological *n* = 6 and ± represent SEMs. *p* > 0.05 using a two-way ANOVA with Greenhouse–Geisser correction.

Time (Days)	Mean Body Weight (g)			
	*Mdx* + Vehicle	*Mdx* + PK007	Control + Vehicle	Control + PK007
1	9.73 ± 0.42	9.88 ± 0.58	9.49 ± 0.53	10.61 ± 0.34
2	9.37 ± 0.48	9.78 ± 0.48	9.78 ± 0.60	11.23 ± 0.43
3	9.33 ± 0.50	9.77 ± 0.42	10.17 ± 0.59	11.68 ± 0.46
4	9.67 ± 0.35	10.00 ± 0.37	10.83 ± 0.57	12.10 ± 0.63
5	9.93 ± 0.35	10.35 ± 0.32	11.38 ± 0.58	12.68 ± 0.52
6	10.50 ± 0.50	10.80 ± 0.35	11.87 ± 0.65	12.82 ± 0.74
7	10.52 ± 0.89	11.77 ± 0.33	12.48 ± 0.63	13.43 ± 0.65
8	11.97 ± 0.62	12.63 ± 0.37	12.98 ± 0.65	13.77 ± 0.68
9	12.60 ± 0.59	13.63 ± 0.55	13.73 ± 0.69	14.58 ± 0.70
10	13.00 ± 0.51	14.05 ± 0.46	14.40 ± 0.71	15.45 ± 0.71

**Table 2:** Tabulated values of mean grip strength of treated and untreated *mdx* mice. Shown are the daily mean grip strength in newtons (N) for vehicle-treated, PK007-treated *mdx* mice, PK007-treated control, and vehicle-treated control mice. Biological *n* = 6 and ± represent SEMs.

Time (Days)	Grip Strength in Newtons (N)			
	*Mdx* + Vehicle	*Mdx* + PK007	Control + Vehicle	Control + PK007
1	0.237 ± 0.019	0.302 ± 0.017	0.368 ± 0.023	0.340 ± 0.025
10	0.372 ± 0.019	0.650 ± 0.030	0.640 ± 0.042	0.587 ± 0.022

## Data Availability

The data generated for the study are available on request to the corresponding author.

## Supplementary Materials

The following are available online at https://www.mdpi.com/article/10.3390/life11090994/s1, Figure S1: Potency of PK007 and HQL-79 in HPGDS enzyme assay, Figure S2: Example of a score sheet to assess the wellbeing of mdx mice when treated with PK007 or Vehicle, Figure S3: Pharmacokinetics of PK007 in male Sprague Dawley rats following a single oral dose at 10 mg/kg, Table S1: Safety panel screen of PK007 on 44 targets, recommended by major pharmaceutical companies, measuring activity (as % inhibition)—performed at Eurofins Cerep-Panlabs, Taiwan, Table S2: Summary of statistical levels.
