# Supplementary material for: Hematopoietic Prostaglandin D Synthase Inhibitor PK007 Decreases Muscle Necrosis in DMD mdx Model Mice
Source: Life (Basel). 2021 Sep 21;11(9):994. doi: 10.3390/life11090994 (PMC8469723; doi:10.3390/life11090994)
Supplement: Supplementary file 1 [file life-11-00994-s001.zip › life-1322563-supplementary.pdf]

Supplementary Table S1: Safety panel screen of PK007 on 44 targets, recommended by major pharmaceutical companies, measuring activity (as % inhibition) – performed at Eurofins Cerep-Panlabs, Taiwan.

**Compound: PK007**						
Cat #	Assay Name	Batch	Spec.	Rep.	Conc.	% Inhibition
104010	Cholinesterase, Acetyl, ACS	438345	hum	2	10 µM	40
116030	Cyclooxygenase COX-1	438269	hum	2	10 µM	-15
118010	Cyclooxygenase COX-2	438270	hum	2	10 µM	-5
140010	Monoamine Oxidase MAO-A	438258	hum	2	10 µM	8
152300	Phosphodiesterase PDE3A	438260	hum	2	10 µM	-2
154420	Phosphodiesterase PDE4D2	438261	hum	2	10 µM	1
176020	Protein Tyrosine Kinase, LCK	438201	hum	2	10 µM	24
200610	Adenosine A2A	438217	hum	2	10 µM	15
203100	Adrenergic α1A	438284	rat	2	10 µM	1
203630	Adrenergic α2A	438239	hum	2	10 µM	5
204010	Adrenergic β1	438227	hum	2	10 µM	2
204110	Adrenergic β2	438210	hum	2	10 µM	1
206000	Androgen (Testosterone)	438399	hum	2	10 µM	22
214600	Calcium Channel L-Type, Dihydropyridine	438340	rat	2	10 µM	22
217030	Cannabinoid CB1	438347	hum	2	10 µM	-2
217100	Cannabinoid CB2	438347	hum	2	10 µM	8
218030	Cholecystokinin CCK1 (CCKA)	438289	hum	2	10 µM	12
219500	Dopamine D1	438231	hum	2	10 µM	-10
219700	Dopamine D2S	438256	hum	2	10 µM	19
224010	Endothelin ETA	438402	hum	2	10 µM	-5
226600	GABAA, Flunitrazepam, Central	438210	rat	2	10 µM	10
232030	Glucocorticoid	438291	hum	2	10 µM	14
232810	Glutamate, NMDA, Agonism	438304	rat	2	10 µM	3
239610	Histamine H1	438360	hum	2	10 µM	5
239710	Histamine H2	438227	hum	2	10 µM	-8
252610	Muscarinic M1	438212	hum	2	10 µM	-7
252710	Muscarinic M2	438213	hum	2	10 µM	-3
252810	Muscarinic M3	438214	hum	2	10 µM	-8
299031	Nicotinic Acetylcholine α4β2, Cytisine	438311	hum	2	10 µM	8
260130	Opiate δ1 (OP1, DOP)	438239	hum	2	10 µM	5
260210	Opiate κ (OP2, KOP)	438296	hum	2	10 µM	14
260410	Opiate µ (OP3, MOP)	438296	hum	2	10 µM	11
265910	Potassium Channel hERG, [3H] Dofetilide	438276	hum	2	10 µM	14
265510	Potassium Channel [KA]	438293	rat	2	10 µM	-5
271110	Serotonin (5-Hydroxytryptamine) 5-HT1A	438367	hum	2	10 µM	7
271230	Serotonin (5-Hydroxytryptamine) 5-HT1B	438254	hum	2	10 µM	9
271650	Serotonin (5-Hydroxytryptamine) 5-HT2A	438299	hum	2	10 µM	10
271700	Serotonin (5-Hydroxytryptamine) 5-HT2B	438235	hum	2	10 µM	67
271910	Serotonin (5-Hydroxytryptamine) 5-HT3	438299	hum	2	10 µM	7
279510	Sodium Channel, Site 2	438310	rat	2	10 µM	48
220320	Transporter, Dopamine (DAT)	438233	hum	2	10 µM	-11
204410	Transporter, Norepinephrine (NET)	438229	hum	2	10 µM	15
274030	Transporter, Serotonin (5- Hydroxytryptamine)	438305	hum	2	10 µM	-10
287530	Vasopressin V1A	438470	hum	2	10 µM	-12

Foot note: For receptor assays, compound binding was calculated as percent inhibition of the binding of radioactively labelled ligand specific for each target. For enzyme assays, the inhibition effect was calculated as percent inhibition of control enzyme activity.

Supplementary Table S2: Summary of statistical levels

	Variable	Test	Mean	SEM	*n*	*p* value
Figure 1	Body Weight (Day 1 and Day 10)	2 Way ANOVA	*mdx* Vehicle: 9.73 g and 13 g<br>*mdx* PK007: 9.88 g and 14.05 g<br>WT-Vehicle: 9.49 g and 14.0 g<br>WT-PK007: 10.61g and 15.45 g	*mdx* Vehicle: 1.02 g and 1.24 g<br>*mdx* PK007: 1.42 g and 1.73 g<br>WT-Vehicle: 1.30 g and 1.73 g<br>WT-PK007: 0.85 g and 1.74 g	6	p>0.05
Figure 2	Grip Strength (Day 1 and Day 10)	2 Way ANOVA	*mdx* Vehicle: 0.237 N and 0.372 N<br>*mdx* PK007: 0.302 N and 0.650 N<br>WT-Vehicle Treated: 0.368 N and 0.640 N<br>WT-PK007 Treated: 0.340 N and 0.587 N	*mdx* Vehicle: 0.019 N and 0.019 N<br>*mdx* PK007: 0.017 N and 0.030 N<br>WT-Vehicle: 0.023 N and 0.042 N<br>WT-PK007: 0.025 N and 0.022 N	6	***<0.0001
Figure 3	Regenerating Muscle fibres (TA)	2 Way ANOVA	*mdx* Vehicle: 73.29%<br>*mdx* PK007: 54.66%<br>WT-Vehicle: 1.60%<br>WT-PK007: 1.07%	*mdx* Vehicle: 4.89%<br>*mdx* PK007: 5.89%<br>WT-Vehicle: 0.16%<br>WT-PK007: 0.18%	6	* 0.0353<br>***<0.0001
Figure 4	Regenerating Muscle fibres (GA)	2 Way ANOVA	*mdx* Vehicle: 57.72%<br>*mdx* PK007 Treated: 38.33%<br>WT-Vehicle: 1.38%<br>WT-PK007: 1.54%	*mdx* Vehicle: 5.78%<br>*mdx* PK007: 4.66%<br>*mdx* WT-Vehicle: 0.12 %<br>*mdx* WT-PK007: 0.21%	6	** *0.0036*<br>***<0.0001
Figure 5	Fibrosis TA muscle	2 Way ANOVA	*mdx* Vehicle: 9.82%<br>*mdx* PK007: 3.12%<br>WT-Vehicle: 0.77%<br>WT-PK007: 0.59%	*mdx* Vehicle: 7.41%<br>*mdx* PK007: 3.60%<br>WT-Vehicle: 1.03%<br>WT-PK007: 0.27%	6	*0.0376<br>**0.002<br>**0.0025
Figure 6	Fibrosis GA Muscle	un-paired *t*-test	*mdx* Vehicle: 5.90%<br>*mdx* PK007: 5.58%	*mdx* Vehicle: 4.44%<br>*mdx* PK007: 2.61%	6	0.8826
Figure 7	CK-MM	un-paired *t*-test	*mdx* Vehicle: 0.002705 mU/ml<br>*mdx* PK007: 0.001094 mU/ml	N/A	5/6	0.0196

Supplementary Figure S1: Potency of PK007 and HQL-79 in HPGDS enzyme assay

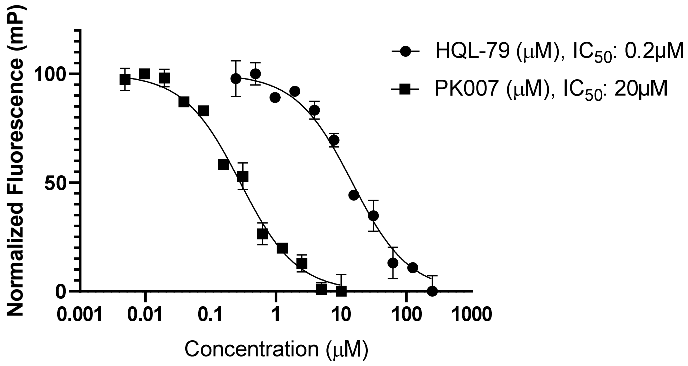

Supplementary Figure S2: Example of a score sheet to assess the wellbeing of *mdx* mice when treated with PK007 or Vehicle:

Experimental/ Treatment Group:

Date: **Time:** **AEC Number:** **Name of person scoring:**

Name of Supervisor/Chief Investigator: **Contact Telephone Number:** **After Hours:**

**Indicators**	**Scoring of independent variables:**	**Score of each animal in group**					
**Date**							
**General Health**<br>Eating	0. drinking and eating well<br>1. change in eating or drinking habit<br>2. inappetance.<br>3. not eating/drinking, severely dehydrated						
Locomotion	0. walking normally<br>1. limping, stiffness<br>2. swollen limbs<br>3. severely restricted mobility						
Behaviour:	0. normal<br>1. away from littermates<br>2. aggressive or huddled in corner<br>3. severe distress						
**Appearance**	0. Normal<br>1. ruffled fur<br>2. animal appears depressed , hunched, reluctant to move)<br>3. animal appears severely depressed.						
**Weight loss**	0. normal<br>1. 5 – 9%<br>2. 10-14%<br>3. ≥15%						

Supplementary Figure S3: Pharmacokinetics of PK007 in male Sprague Dawley rats following a single oral dose at 10 mg/kg.

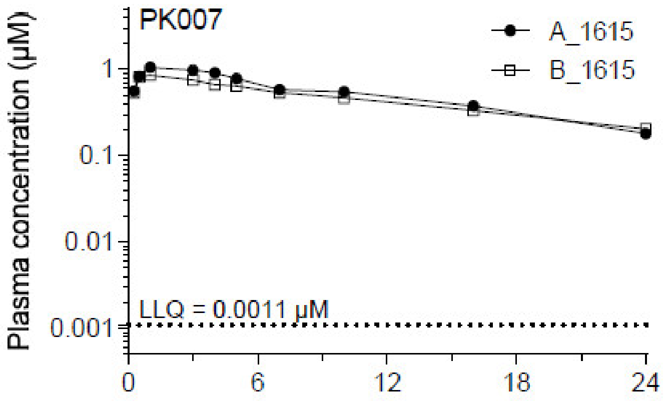

Parameter	PK007
Potency (µM)	0.2
$T_{1/2}$ (hrs)	11.0
C_{max} (µM)	1.0
$AUC_{0\text{-}inf}$(h*µM)	15.0
